# The D2 Dopamine Receptor Interferes With the Protective Effect of the A_2A_ Adenosine Receptor on TDP-43 Mislocalization in Experimental Models of Motor Neuron Degeneration

**DOI:** 10.3389/fnins.2018.00187

**Published:** 2018-03-20

**Authors:** Chia-You Lai, Yu-Ju Liu, Hsing-Lin Lai, Hui-Mei Chen, Hung-Chi Kuo, Yu-Ping Liao, Yijuang Chern

**Affiliations:** ^1^Graduate Institute of Life Sciences, National Defense Medical Center, Taipei, Taiwan; ^2^Institute of Biomedical Sciences, Academia Sinica, Taipei, Taiwan; ^3^Institute of Cellular and Organismic Biology, Academia Sinica, Taipei, Taiwan

**Keywords:** TDP-43, A_2A_ adenosine receptor, D2 dopamine receptor, cAMP, PKA motor neurons, ALS

## Abstract

The A_2A_ adenosine receptor (A_2A_R) and D2 dopamine receptor (D_2_R) are two G-protein-coupled receptors that can form dimers and negatively regulate their partners. TAR DNA-binding protein (TDP-43) is a nuclear protein that has been implicated in amyotrophic lateral sclerosis (ALS). Mislocalization of TDP-43 from the nucleus to the cytoplasm is an early step of TDP-43 proteinopathy. Our previous studies indicated that A_2A_R is a potential drug target for ALS because treatment with an A_2A_R agonist (JMF1907; a T1-11 analog) prevents reactive oxygen species (ROS)-induced TDP-43 mislocalization in a motor neuron cell line (NSC34) and delays motor impairment in a TDP-43 transgenic ALS mouse model. Here, we set out to assess whether activation of D_2_R interferes with the beneficial effects of an A_2A_R agonist on motor neurons. We first demonstrated that A_2A_R and D_2_R are both located in motor neurons of mouse and human spinal cords and human iPSC-derived motor neurons. Expression of A_2A_R and D_2_R in NSC34 cells led to dimer formation without affecting the binding affinity of A_2A_R toward T1-11. Importantly, activation of D_2_R reduced T1-11-mediated activation of cAMP/PKA signaling and subsequent inhibition of TDP-43 mislocalization in NSC34 cells. Treatment with quinpirole (a D_2_ agonist) blunted the rescuing effect of T1-11 on TDP-43 mislocalization and impaired grip strength in a mouse model of ALS. Our findings suggest that D_2_R activation may limit the beneficial responses of an A_2A_R agonist in motor neurons and may have an important role in ALS pathogenesis.

## Introduction

Amyotrophic lateral sclerosis (ALS) is a progressive motor neuron disease that most severely damages the motor cortex, brain stem, and spinal cord. Approximately 5–10% of ALS cases are familial, with the average onset is 47-52 years of age (Kiernan et al., 2011). TAR-DNA-binding protein-43 (TDP-43) is a nuclear protein that regulates gene transcription and mRNA processes (Cohen et al., 2011). Ample evidence suggests that abnormal cellular distribution, cleavage, and inclusion formation in motor neurons of the spinal cord are closely associated with ALS (Neumann et al., 2006; Chen-Plotkin et al., 2010). More than 40 mutation sites (including A315T, M337V) were observed in a glycine-rich domain located in the carboxyl-terminus of TDP-43 that are closely associated with familial ALS (Gitcho et al., 2008; Cairns et al., 2010). We have previously reported that mislocalization of TDP-43 from the nucleus to the cytoplasm may be one of the initial steps that occur during ALS pathogenesis (Liu et al., 2015a). It is of great interest to note that such an abnormal distribution of TDP-43 in the cytoplasm elevates oxidative stress in a feedforward manner, which is closely associated with the pathology of ALS (Ayala et al., 2011; Dewey et al., 2011; D'Amico et al., 2013). Although tremendous effort has been devoted to developing therapeutic treatments for ALS, only two drugs with limited effectiveness are currently available for ALS (Riluzole and Radicava). Riluzole targets the glutamatergic system (Doble, 1996), while Radicava is a free radical scavenger that protects motor neurons from oxidative stress (Mullard, 2017).

AMP-activated protein kinase (AMPK) is a major player in cellular energy homeostasis and can be regulated by reactive oxygen species (ROS) (Ju et al., 2014). The holoenzyme of AMPK is composed of a catalytic α subunit and two regulatory β and γ subunits. In addition to ROS, AMPK primarily is regulated by the cellular AMP:ATP ratio and is activated by multiple upstream kinases that phosphorylate AMPK-α subunits on threonine 172 (Cardaci et al., 2012). Conversely, we and several laboratories have demonstrated that AMPK can be phosphorylated by protein kinase A (PKA) at α1–Ser^173^ and α1–Ser^485^, leading to inactivation (Hurley et al., 2006; Djouder et al., 2010; Ju et al., 2011). Accumulated evidence from several experimental models of ALS [such as the superoxide dismutase 1 mutant mouse (Lim et al., 2012) and a motor neuron cell line (Liu et al., 2015a)] suggests that abnormal over-activation of AMPK in motor neurons is detrimental for survival. We previously demonstrated that activation of the A_2A_ Adenosine receptor (A_2A_R) inhibits AMPK through a cAMP/PKA-dependent pathway in experimental models of Huntington's disease (HD) and ALS (Ju et al., 2011; Liu et al., 2015a,b). T1-11 is a dual-function adenosine compound with moderate affinity toward A_2A_R and an adenosine transporter (equilibrative nucleoside transporter 1, ENT1) that has been demonstrated to ameliorate motor degeneration in HD mice (Huang et al., 2011) and SCA3 (Chou et al., 2015) as well as to improve neurovisceral symptoms in Niemann-Pick type C disease (Ferrante et al., 2017). Chronic treatments with a T1-11 analog (JMF1907) that has similar properties also alleviated the impairment of motor function in mouse models of HD and ALS (Liu et al., 2015a; Kao et al., 2017). These studies suggest that A_2A_R is a potential therapeutic target for several degenerative diseases, including ALS.

A_2A_R is a Gαs-protein-coupled receptor (Chen et al., 2014) that activates adenylyl cyclases and produces cAMP upon stimulation (Chang et al., 1997). A_2A_R has long been implicated in ALS because stimulation of A_2A_R is known to transactivate the BDNF receptor (TrkB) in the absence of BDNF in motor neurons (Yanpallewar et al., 2012). In addition, the A_2A_R-mediated cAMP/PKA pathway protects motor neurons from the toxicity of AMPA (Komaki et al., 2012). We are particularly interested in the D2 dopamine receptor (D_2_R) because it is a Gαi-coupled receptor that suppresses adenylyl cyclases and inhibits production of cAMP. Most importantly, D_2_R has been demonstrated to directly interact with A_2A_R (Canals et al., 2003), and a reciprocal antagonistic interaction between A_2A_R and D_2_R has been well-documented. For example, stimulation of D_2_R diminishes the ability of A_2A_R to bind to its agonists and activate adenylyl cyclases (Fernández-Dueñas et al., 2012, 2013). The existence of A_2A_R/D_2_R heterodimers has been observed in the striatum of both rodents (Ferre et al., 1994) and humans (Díaz-Cabiale et al., 2001) as well as in cell lines exogenously expressing both A_2A_R and D_2_R (Kull et al., 1999; Hillion et al., 2002; Canals et al., 2003). In motor neurons of the lumbar spinal cord, D_2_R is the most highly expressed dopamine receptor (Zhu et al., 2007). We therefore aimed to investigate whether D_2_R forms a functional complex with A_2A_R in motor neurons and interferes with A_2A_R/cAMP/PKA-mediated rescue of TDP-43 mislocalization in ALS. In the present study, we report that D_2_R is colocalized with A_2A_R in motor neurons of the spinal cord and diminishes the cAMP/PKA signal that plays a critical role in the A_2A_R-mediated neuroprotective effects on TDP-43 mislocalization and grip strength in ALS.

## Materials and methods

### Reagents and antibodies

Adenosine deaminase (ADA), protease inhibitor and phosSTOP were obtained from Roche (Basel, Switzerland). The anti-A_2A_R monoclonal antibody (mAb, clone 7F6-G5-A2) was purchased from Santa Cruz Biotechnology (Santa Cruz, CA, USA). The human anti-A_2A_R antibody was generated against the C terminus of human A_2A_R (NH2-PPGLDDPLADGAG-COOH). The mouse A_2A_R was raised against the C terminus of mouse A_2A_R (NH2-TQEHQEGQEHPGLG-COOH) (GenScript; Piscataway, NJ, USA). The human anti-D_2_R antibody and the anti-ChAT antibody were purchased from Millipore (Bedford, MA, USA). The guinea pig anti-D_2_R antibody was obtained from Frontier Institute co. (Hokkaido, Japan). The mouse/human and human anti-TDP-43 mAbs were obtained from Abcam (Cambridge; MA, USA) and Abnova (Taipei, Taiwan), respectively. The anti-phospho-AMPKα (Thr172) antibody was purchased from Cell Signaling Technology (Danvers, MA, USA). The anti-AMPKα1 antibody was obtained from GeneTex (Irvine, CA, USA). The anti-β actin pAb, the anti-flag pAb, protein A beads and the proximity ligation assay (PLA) kit were purchased from Sigma (St Louis, MO, USA). The anti-V5 mAb was purchased from Invitrogen (Carlsbad, CA, USA). AlexaFluor®−488, −568, −647, and rhodamine-phalloidin were obtained from Molecular Probes (Eugene, OR, USA). Quinpirole, quinelorane and L741,626 were purchased from Tocris Bioscience (Bristol, UK). The antigen retrieval reagent was purchased from Dako (Kyoto, Japan). The cAMP assay kit and the tyramide signal amplification (TSA) kit were obtained from PerkinElmer (Massachusetts, US). The PKA assay kit was purchased from Enzo Life Sciences (Framingdale, NY, USA).

### Constructions

The full-length D_2_R and A_2A_R constructs were amplified from mouse striatum cDNAs by polymerase chain reaction (PCR; primers for D_2_R: 5′-GCAAGCTTGCCACCATGGATCCACTGAACCTG-3′ and 5′-TCTCGAGGCAGTGCAGGATCTTCATGAAGGC-3′; primers for A_2A_R: 5′-GCAGTTGCTAAGCTTGCCACCATGGGCTCCTCGGTG-3′ and 5′-CCGGGATCCTCTAGAGGAAGGGGCAAACTC-3′), and subcloned into pcDNA3.1/V5-His-TOPO (Invitrogen) and p3X FLAG-CMV14 (Sigma), respectively. The sequences of the resultant constructs were validated by DNA sequences.

### Human spinal cord sections

Spinal cord sections of non-ALS and ALS subjects were obtained from the National Institute of Child Health and Development (NICHD) Brain and Tissue Bank for Developmental Disorders (Baltimore, MD, USA). This study was approved by the Institutional Review Board (IRB) on Biomedical Science Research/IRB-BM, Academia Sinica (AS-IRB-BM-11071). Immunohistochemistry (IHC) staining was performed as previously reported with slight modifications (Asson-Batres and Smith, 2006; Ju et al., 2011). Briefly, human spinal cord sections (5 μm) were pretreated with an antigen retrieval reagent (Dako; pH 9.0) at 95°C for 30 min, washed with 0.1 M Na-PBS, and permeabilized with 0.1 M Na-PBS containing 0.2% Triton X-100 for 10 min. To block endogenous peroxidase activity, the sections were incubated with 0.1 M Na-PBS containing 1.5% H_2_O_2_ and 10% methanol for 10 min and then blocked with 4% bovine serum albumin (BSA) for 2 h at room temperature (RT). The slide was incubated with the anti-ChAT antibody for 24 h at 4°C, followed by a 2 h incubation with a secondary antibody conjugated with AlexaFlour®568. Slides were then washed with 0.1 M Na-PBS and blocked with 4% BSA for 2 h, followed by incubation with the anti-hA_2A_R antibody for 24 h at 4°C and a 2 h incubation with a biotinylated corresponding secondary antibody. After extensive washes, avidin-biotinylated-HRP was added to the slides and incubated for 90 min, followed by an incubation with a TSA-containing fluorescence reagent for 10 min. Slides were then washed with 0.1 M Na-PBS containing 1% H_2_O_2_ to the remove unconjugated horseradish peroxidase (Asson-Batres and Smith, 2006), blocked with 4% BSA for 2 h, incubated with the anti-hD_2_R pAb for 24 h at 4°C and then incubated with a biotinylated secondary antibody for another 2 h. Avidin-biotinylated-HRP was applied to the slides for another 90 min. Slides were incubated with the conjugated TSA fluorescence reagent for 10 min. After extensive washes, slides were mounted with a mounting medium containing DAPI (Biotium; Fremont, CA, USA). Images were acquired using confocal microscopy (LSM 700stage, Carl Zeiss; Oberkochen, Germany). For negative controls, slides were stained following the same procedures as described above, except that only the anti-hA_2A_R antibody or 4% BSA was used for staining for 24 h at 4°C. Slides were washed with 0.1 M Na-PBS containing 1% H_2_O_2_, blocked with 4% BSA for 2 h, incubated with 4% BSA or an anti-hD_2_R antibody for 24 h at 4°C, followed by the TSA immunostaining procedure described above. Mouse spinal cord sections were stained with the anti-A_2A_R antibody first and then stained with an anti-D_2_R antibody using the TSA amplification procedure described above. Images were analyzed using confocal microscopy (LSM 700stage, Carl Zeiss; Oberkochen, Germany).

### Preparation of human motor neurons

Human motor neurons were derived from human induced pluripotent stem cells (hiPSCs). This study was approved by IRB on Biomedical Science Research/ IRB-BM, Academia Sinica (AS-IRB-BM-17002). The protocol for motor neuron generation was performed as previously described (Du et al., 2015). Briefly, motor neuron progenitors (MNPs) were generated from hiPSCs using a combination of small molecules, 3 μM CHIR99021, 2 μM DMH1, and 2 μM SB431542, for 6 days. MNPs were maintained in culture medium containing 1 μM CHIR99021, 2 μM DMH1, 2 μM SB431542, 1 μM RA, and 1 μM Purmorphamine (Pur) for an additional 6 days. To induce MN differentiation, MNPs were cultured in a medium with 1 μM RA and 1 μM Pur for additional 6 days. Finally, MNs were plated on Matrigel-coated plates and cultured with 0.1 μM RA, 0.5 μM Pur, 0.1 μM compound E, 1 μM cAMP, 10 ng/ml BDNF, 10 ng/ml GDNF, 10 ng/ml CNTF, and 10 ng/ml IGF for further 12 d. MNPs were fixed with 4% paraformaldehyde (PFA) for 30 min, washed three times with PBS for 5 min and permeabilized with 0.05% NP40 in PBS for 10 min. Next, they were blocked with 3% BSA in PBS, incubated with an anti-ChAT antibody for 24 h at 4°C, and incubated with a secondary antibody conjugated with AlexaFlour®568 for 2 h. After extensive washes, MNP cells were blocked with BSA for 2 h and incubated with the indicated primary antibodies for another 48 h at 4°C, followed by incubation with indicated secondary antibodies conjugated with AlexaFlour®−488 and −647 and mounted in a mounting solution containing DAPI. Images were analyzed using confocal microscopy (LSM 700stage, Carl Zeiss; Oberkochen, Germany).

### Cell culture

The motor neuron-like cell line, NSC34, was kindly provided by Dr. Cashman (Cashman et al., 1992). Cells were cultured in Dulbecco's modified Eagle's medium with 10% fetal bovine serum and 1% penicillin-streptomycin (Invitrogen) under 5% CO_2_ at 37°C.

### Immunofluorescence

NSC34 cells were pretreated with ADA (1 U/ml) for 4 h to remove endogenous adenosine and were stimulated with the indicated drugs for another 4 h at 37°C to assess the distribution of TDP-43 by immunofluorescence staining. NSC34 cells were first fixed with methanol (kept at −20°C) for 10 min and washed three times with PBS for 5 min. Cells were permeabilized with 0.05% NP40 in PBS for 10 min, blocked with 3% normal goat serum (NGS) or BSA in PBS and incubated with the indicated primary antibody for 18–22 h at 4°C, and this was followed by incubation with the indicated secondary antibody conjugated with AlexaFlour®−488 or −568 and mounting with a mounted solution containing DAPI. Images were analyzed using confocal microscopy (LSM 700stage, Carl Zeiss; Oberkochen, Germany) and analyzed blinded.

Sections from the thoracic region of the mouse spinal cord were prepared as previously reported (Liu et al., 2015a). In brief, tissue slices (30 μm) were permeabilized with 0.1 M Na-PBS containing 0.2% Triton X-100, three times (10 min for each wash). To block endogenous peroxide activity, sections were incubated with 0.1 M Na-PBS containing 1.5% H_2_O_2_ and 10% methanol for 10 min, blocked with 3% BSA for 2 h, incubated with the desired primary antibody for 36–40 h at 4°C, and then incubated with AlexaFlour®−488 or −568 for 2 h. Images were obtained using confocal microscopy (LSM 700stage, Carl Zeiss; Oberkochen, Germany) and analyzed blinded. At least 3 different animals, with 16 images and 40 motor neurons for each animal, were scored for each condition.

### Proximity ligation assay (PLA)

NSC34 cells were transiently transfected with V5-mD_2_R and FLAG-mA_2A_R using Lipofectamine™ 2000 (Invitrogen) for 48 h and fixed with 4% PFA for 30 min, followed by extensive washes. PLA was carried out as detailed earlier (Trifilieff et al., 2011). Briefly, slides were incubated with the desired primary antibodies for 48 h at 4°C, followed by incubation with PLA-conjugated secondary antibodies for 60 min at 37°C and extensive washes. Next, slides were incubated with the ligase mixture for 30 min at 37°C. PLA signals were amplified for 100 min at 37°C. Images were acquired using a confocal microscope (LSM 700stage, Carl Zeiss; Oberkochen, Germany).

### Immunoprecipitation and western blotting

Immunoprecipitation was carried out as previously reported (Fernández-Dueñas et al., 2012; Wu et al., 2013). NSC34 cells were solubilized in lysis buffer (50 mM Tris-HCl, pH 7.4, 100 mM NaCl, 1% Triton X-100, 0.5% sodium deoxycholate, 0.2% sodium dodecyl sulfate, 1 mM EDTA and protease inhibitor and phosSTOP) on ice for 30 min and centrifuged at 13,200 rpm for 30 min. The supernatant (3 mg) was treated with 1 μg of the primary antibody for 24 h at 4°C and incubated with 40 μl of protein A beads overnight at 4°C. The immune complex was washed twice with lysis buffer, twice with 0.1% lysis buffer diluted in Tris-buffered saline (TBS; 50 mM Tris-HCl, pH 7.4, 100 mM NaCl) and once with TBS alone. Immunoprecipitation complexes were dissolved in 40 μl of 4x sample buffer, separated via SDS-PAGE, transferred to a PVDF (Millipore) membrane, and probed with the indicated primary antibodies. Immune signaling was analyzed by the ECL reagent (PerkinElmer).

NSC34 cells were pretreated with ADA (1 U/ml) for 4 h to remove endogenous adenosine and were stimulated with the indicated drug(s) for an additional 4 h at 37°C to measure AMPK activity.

### cAMP assay

NSC34 cells were pretreated with ADA (1 U/mL) to remove endogenous adenosine for 4 h at 37°C, followed by treatment with the indicated drug(s) for 15 min at 37°C. Cells were washed three times with ice-cold Locker's solution (150 mM NaCl, 5.6 mM KCl, 5 mM glucose, 1 mM MgCl_2_, and 1 mM EDTA, pH 7.4) and incubated with 0.1 N HCl for 10 min to extract cellular cAMP. The cAMP content was determined by a ^3^H-cAMP assay kit following the manufacturer's protocol (PerkinElmer).

### PKA activity

NSC34 cells or the lumbar regions of spinal cords were homogenized in a non-denaturing buffer (20 mM Tris, 137 mM NaCl, 1% NP40, 2 mM EDTA, 1 mM sodium orthovanadate, 50 mM sodium fluoride, 1 mM phenylmethane-sulphonylfluoride (PMSF), 40 μM leupeptin, protease inhibitor and phosSTOP Cocktail) by 20 strokes with mini blue douncers, followed by centrifugation (16,000 × g for 20 min) at 4°C to harvest the supernatants. The PKA activity of the indicated lysate (0.5 μg of NSC34 cells or 0.1 μg of spinal cords) was determined using a PKA assay kit following the manufacturer's protocol.

### Animals and drug(s) administration

A315T TDP-43 ((Prnp-TARDBP^*^A315T) 23Jlel/J) (Stallings et al., 2010) mice and their littermate control mice ((C57BL/6 × SJL) F1) were purchased from Jackson Laboratory (Bar Harbor, ME, USA) and bred in the animal core of IBMS at Academia Sinica (Taipei, Taiwan). All animal experiments were conducted using protocols approved by the Academia Sinica Institutional Animal Care and Utilization Committee. Offsprings were genotyped by PCR using the forward primer 5′-ATGGGTGGTGGGATGAACTT-3′ and the reverse primer 5′-ATACCCCAACTGCTCTGTAGTGCT-3′.

A315T TDP-43 (Tg) and littermate control (NTG) mice were fed with T1-11 (0.25 mg/ml) (Huang et al., 2011) or vehicle (1% DMSO) in their drinking water. The D_2_R agonist, quinpirole (6 mg/kg; de Haas et al., 2012) or saline was given to animals by a daily intraperitoneal injection from the age of 7 to 10 weeks. Of note, administration of quinpirole caused an immediate hypolocomotor activity in both Tg and NTG mice as reported earlier (Mattingly et al., 1993; Dall'olio et al., 1997). Thus, the analysis of grip strength was always conducted at least 20 h after the last injection of quinpirole. During the 3-week quinpirole treatment, the transient hypolocomotor activity caused by quinpirole administration was consistently observed. No effect of quinpirole on bodyweight was detected.

### Grip strength

The grip strength test was carried out as previously described (Liu et al., 2015a). Grip strength was assessed once a week before the daily injection of quinpirole for 3 weeks. The grip strength of each animal represents the average value of three independent tests and was normalized to the grip strength of each animal at 7 weeks before the 3-week treatment.

### Statistical analysis

All statistical analyses were carried out using version 3.5 of SigmaState (San Jose, California). Unless stated otherwise, statistical analyses were carried out by one-way ANOVA, followed by the Student-Newman-Keuls multiple comparisons *post-hoc* test. Differences at *p* < 0.05 were considered statistically significant.

## Results

### D_2_R forms complexes with A_2A_R in motor neurons

**Figure 1:**
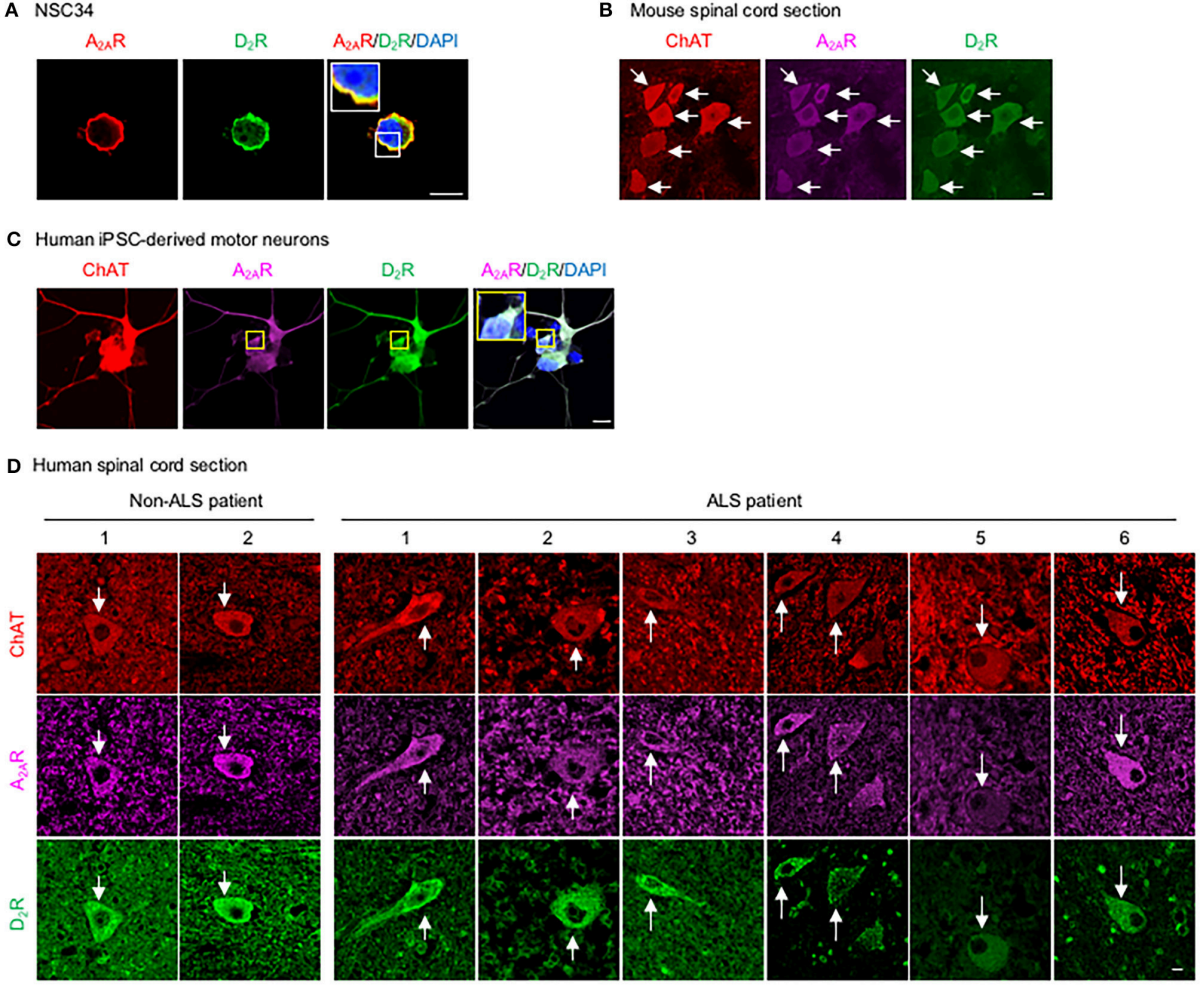
D_2_R is co-localized with A_2A_R in motor neurons. **(A)** Motor neuron-like cells (NSC34) were stained for A_2A_R (red) and D_2_R (green) as well as with a nuclear marker (DAPI, blue). **(B)** Spinal cord sections of mice were stained for A_2A_R (purple) and D_2_R (green) using a TSA-amplified immunofluorescence method. To identify motor neurons in mice, sections were stained for a motor neuron marker (ChAT, red). **(C)** Human iPSC-derived motor neurons were stained for A_2A_R (purple) and D_2_R (green), as well as with a nuclear marker (DAPI, blue) and a motor neuron marker (ChAT, red). **(D)** Human spinal cord sections were stained for A_2A_R (purple) and D_2_R (green) using a TSA-amplified immunofluorescence method. Motor neurons from a human spinal cord were also stained for the motor neuron marker (ChAT, red). Scale bar: 10 μm.

To assess whether D_2_R and A_2A_R are co-expressed in the same population of motor neurons in the spinal cord and whether they functionally interact, we first demonstrated that a mouse motor neuron-like cell line (NSC34) endogenously expressed both D_2_R and A_2A_R (Figure 1A). Motor neurons in the mouse spinal cord, identified by the expression of choline acetyltransferase (ChAT), also contained both D_2_R and A_2A_R, as detected by a TSA-amplified immunofluorescence method (Figure 1B). Consistent with the expression found in murine motor neurons, both D_2_R and A_2A_R were detected in human iPSC- derived motor neurons (Figure 1C). Similarly, both D_2_R and A_2A_R can also be observed in motor neurons of spinal cords of non-ALS and ALS subjects by the TSA-amplified immunofluorescence method (Figure 1D; Table 1). Omitting primary antibodies resulted in no signal (Figure S1). Taken together, D_2_R and A_2A_R are co-localized in mouse and human motor neurons of spinal cords.

**Table 1:** Summary of the demographic data and immunostaining results of human subjects.

**Human spinal cord**	**Age (year)**	**Co-expression of D_2_R and A_2A_R in motor neurons of the spinal cord**
ALS-1	60–65	Yes
ALS-2	70–75	Yes
ALS-3	60–65	Yes
ALS-4	66–70	Yes
ALS-5	55–60	Yes
ALS-6	45–50	Yes
Non-ALS-1	40–45	Yes
Non-ALS-2	40–45	Yes

*Spinal cord sections were obtained from the NICHD Brain and Tissue Bank for Developmental Disorders at the University of Maryland, College Park, MD, USA. Spinal cord sections were analyzed by immunofluorescence staining of D_2_R and A_2A_R, as shown in Figure 1D*.

**Figure 2:**
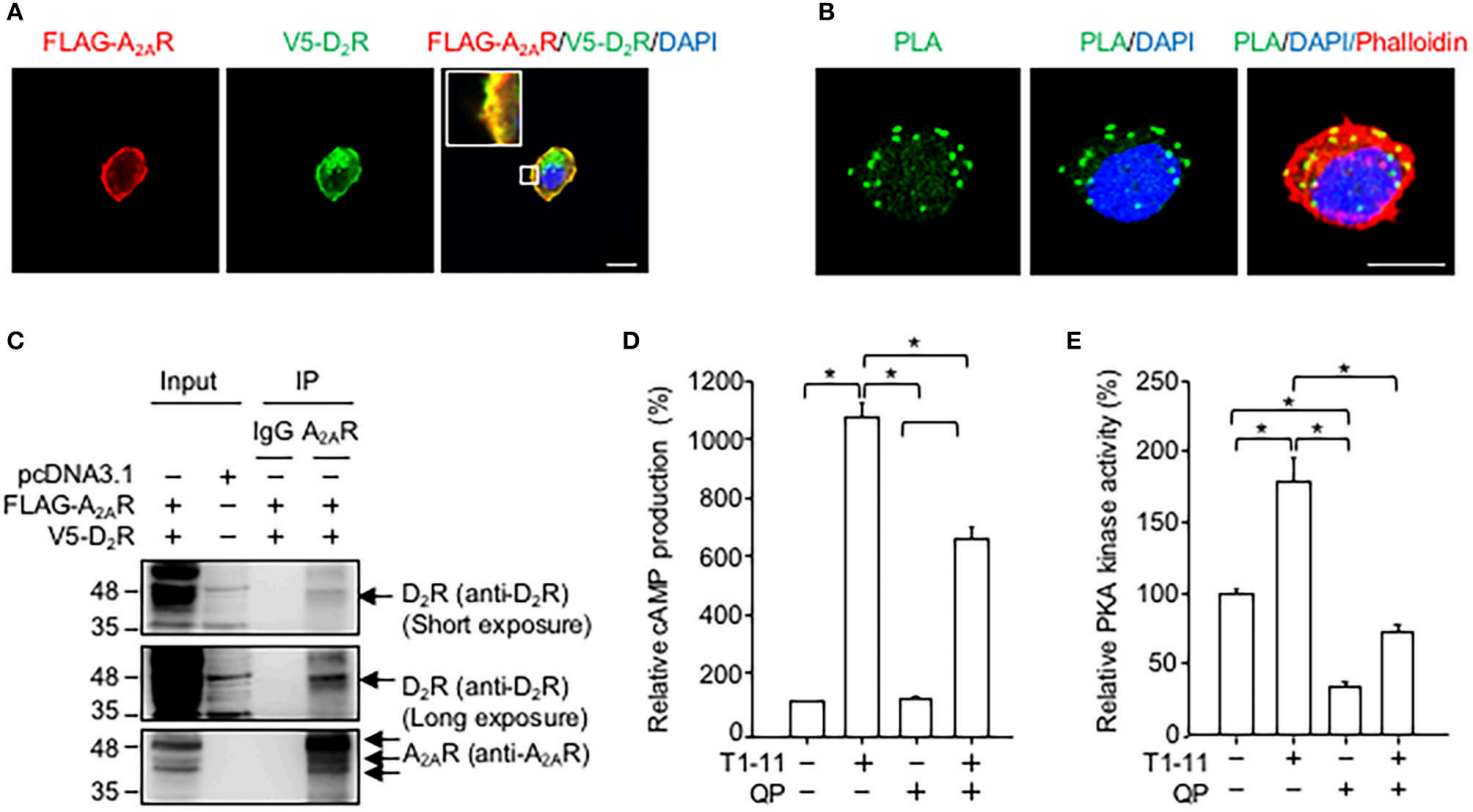
D_2_R forms complexes with A_2A_R in NSC34. **(A)** NSC34 cells were transfected with FLAG-A_2A_R and V5-D_2_R for 48 h. Next, cells were stained with a FLAG antibody (red), a V5 antibody (green) and nuclear marker (DAPI, blue). Scale bar: 10 μm. **(B)** To verify the interaction between FLAG-A_2A_R and V5-D_2_R, cells were stained with a FLAG antibody and a V5 antibody by using the PLA detection method. The cell morphology was analyzed using Rhodamine-phalloidin staining (red). Scale bar: 10 μm. **(C)** NSC34 cells were transfected with the indicated plasmids for 48 h. Next, cells were lysed to examine the interaction between FLAG-A_2A_R and V5-D_2_R by immunoprecipitation (IP) using the indicated antibodies. **(D)** NSC34 cells were incubated with T1-11 (30 μM) in the absence or presence of quinpirole (QP; a D_2_R agonist, 1 μM) for 15 min. Cells were harvested to determine cAMP production. **(E)** NSC34 cells were treated with T1-11 (30 μM) in the absence or presence of QP (1 μM) for 30 min. Next, cells were harvested to determine PKA activity. ^*^p < 0.05, significantly different between the indicated groups. Data are presented as the mean ± SEM of three independent experiments.

Exogenously expressed D_2_R and A_2A_R colocalized in a motor cell line (NSC34, Figure 2A). The results of the PLA revealed that these two receptors were likely to directly interact (Figure 2B) because the PLA signals (green puncta) were clearly visible in NSC34 cells (Figure 2B). No PLA signal was observed when one of the two primary antibodies (i.e., anti-V5 or anti-FLAG antibody) was omitted in the reaction (Figure S2). Moreover, immunoprecipitation assays demonstrated that D_2_R was located in the A_2A_R immunocomplex complex (Figure 2C), while A_2A_R was located in the D_2_R immunocomplex (Figure 2C). Stimulation of A_2A_R with a low affinity agonist (T1-11) enhanced the cellular cAMP levels (Figure 2D) and PKA activity (Figure 2E) in NSC34 cells. Treatment with a D_2_ agonist (quinpirole) reduced the T1-11-evoked cAMP levels (Figure 2D) and PKA activity (Figure 2E). Binding analyses demonstrated that the affinity of T1-11 toward A_2A_R was not altered by D_2_R (the *Ki* values for A_2A_R were 4.0 ± 1.6 μM and 2.7 ± 0.5 μM in the absence or presence of D_2_R, respectively; Table 2). Activation of D_2_R using quinpirole (1 μM) did not affect the binding affinity of T1-11 toward A_2A_R either (the *Ki* values for A_2A_R were 3.8 ± 1.2 and 4.4 ± 0.9 μM in the absence or presence of D_2_R, respectively; Table 2). Collectively, activation of D_2_R negatively regulates A_2A_R-evoked cAMP signaling, without significantly affecting the binding affinity of T1-11 toward A_2A_R.

**Table 2:** Binding properties of T1-11 toward A_2A_R in the absence or presence of D_2_R.

**cDNA**	***K***_**i**_ **values (μM)**	
	**CON**	**QP**
A_2A_R	4.0 ± 1.6	3.8 ± 1.2
A_2A_R + D_2_R	2.7 ± 0.5	4.4 ± 0.9

*NSC34 cells were transfected with A_2A_R in the presence or absence of D_2_R for 44 h, followed by a 4-h incubation with ADA (1 U/ml) at 37°C to remove endogenous adenosine. The membrane lysates were collected and subjected to the receptor binding assay to compete ^3^H-CGS21680 in the absence or presence of QP (1 μM), as described in the Supplemental Method. The data is presented the mean ± SEM of three independent experiments*.

### Activation of D_2_R suppressed A_2A_R-mediated protection of TDP-43 mislocalization in a motor neuron-like cell line (NSC34)

**Figure 3:**
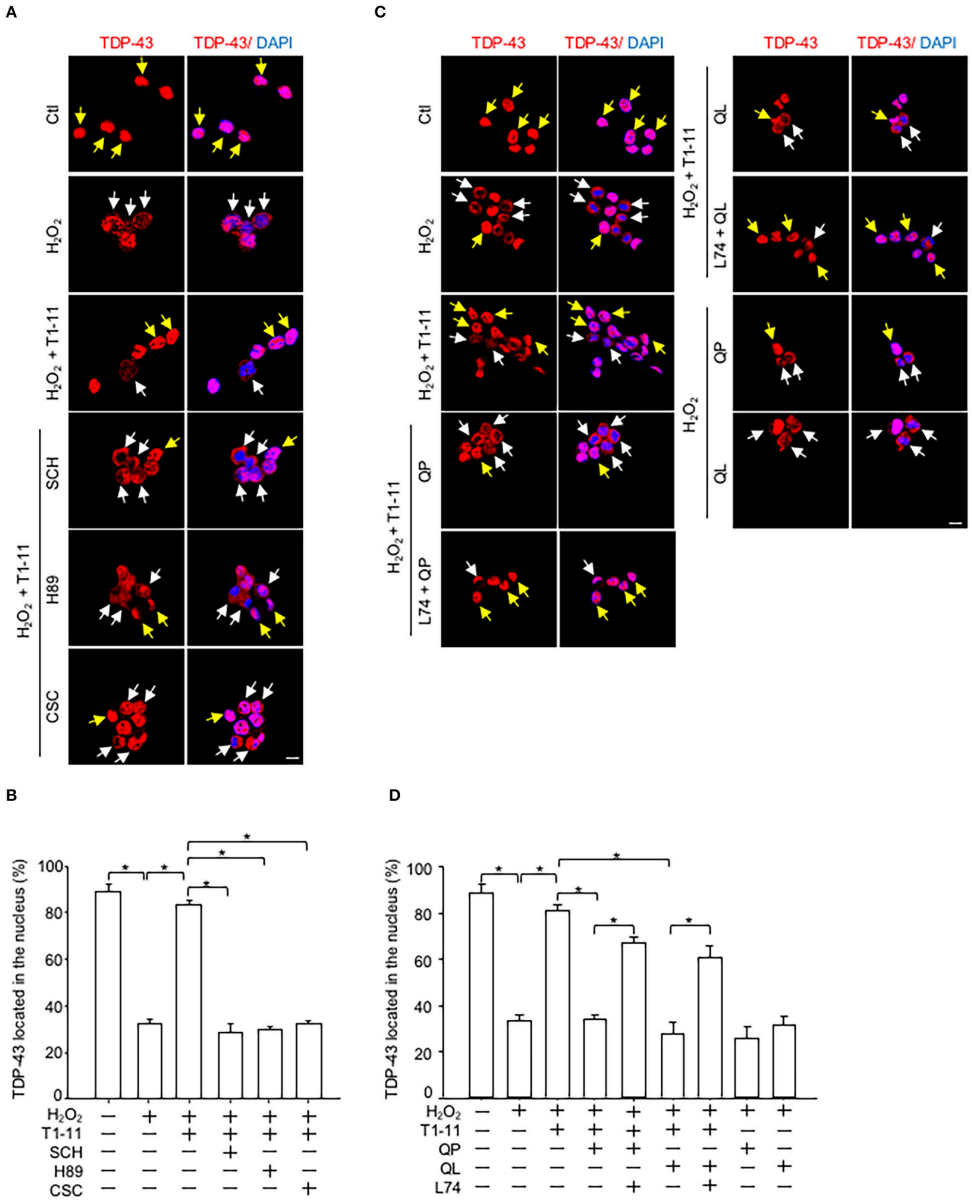
D_2_R activation inhibited the A_2A_R/PKA-mediated protective effects on ROS-induced TDP-43 mislocalization. **(A,B)** NSC34 cells were treated with the indicated drug(s) [H_2_O_2_, 500 μM; T1-11, 30 μM; SCH58261 (an A_2A_R antagonist, 10 μM); H89 (a PKA inhibitor, 5 μM) or CSC (an A_2A_R antagonist, 10 μM)] for 4 h. Localization of TDP-43 (red) was examined by immunofluorescence staining in **(A)**. The quantified results are presented in **(B)**. Data are presented as the mean ± SEM of three independent experiments. **(C,D)** NSC34 cells were incubated with the indicated drug(s) [H_2_O_2_, 500 μM; T1-11, 30 μM; QP (1 μM); quinelorane (QL; a D_2_R agonist; 1 μM) or L741,626 (L74; a selective antagonist of D_2_R, 10 μM)] for 4 h. Localization of TDP-43 (red) was determined by immunofluorescence staining, as shown in **(C)**. The quantified results are shown in **(D)**. Data are expressed as the mean ± SEM of three time experiments. ^*^p < 0.05, significantly different between the indicated groups. The yellow arrow indicates TDP-43 located in the nucleus, and the white arrow indicates TDP-43 mislocalization. Scale bar: 10 μm.

Immunofluorescence staining showed that activation of A_2A_R by T1-11 suppressed oxidative stress-induced TDP-43 mislocalization in NSC34 cells. The effect of T1-11 was mediated by the A_2A_R-PKA pathway because two A_2A_R-selective antagonists [SCH58261, SCH; 8-(3-chlorostyryl) caffeine, CSC] and a PKA inhibitor (H89) all prevented the effect of T1-11 on TDP-43 mislocalization (Figures 3A,B). Importantly, co-stimulation with two D_2_ agonists (quinpirole or quinelorane) hampered the T1-11-mediated protective effects on H_2_O_2_-evoked TDP-43 mislocalization (Figures 3C,D). A selective antagonist of D_2_R (L741,626, L74) eliminated the effects of quinpirole and quinelorane on TDP-43 mislocalization (Figures 3C,D), confirming the involvement of D_2_R.

### Chronic treatment with quinpirole eliminated the beneficial effect of T1-11 in the A315T TDP-43 Tg mice

**Figure 4:**
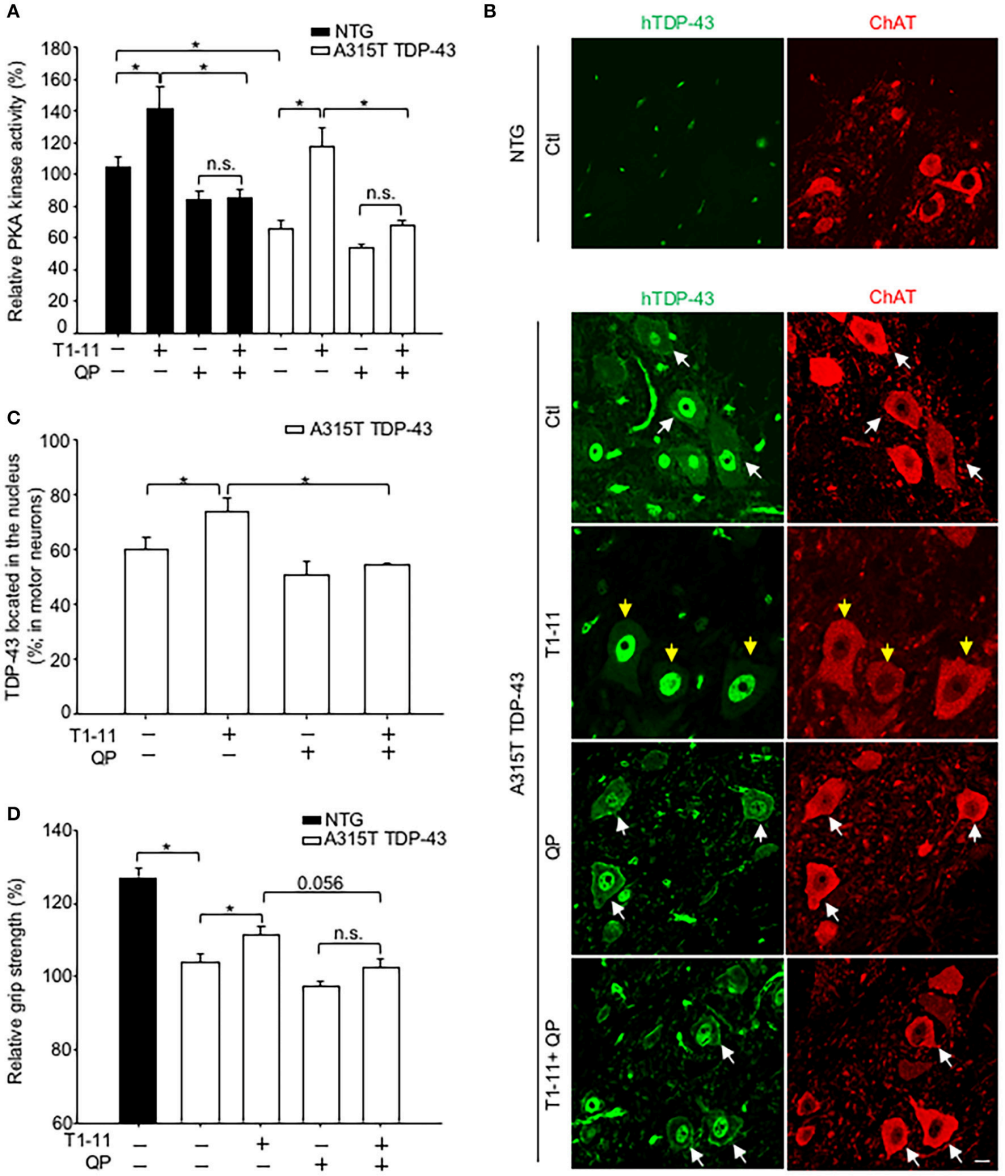
Activation of D_2_R inhibited the A_2A_R-mediated beneficial effects *in vivo*. Transgenic mice (A315T TDP-43) and littermate control mice (non-transgenic mice; NTG) and were treated with T1-11 (0.25 mg/ml) or vehicle (1% DMSO) in the drinking water and co-treated with QP (6 mg/kg) or saline by intraperitoneal injection from the age of 7 weeks (*N* = 5–7). **(A)** Spinal cords from the indicated mice were harvested to examine PKA activity at the age of 10 weeks (*N* = 5–7). **(B,C)** Spinal cord sections from the indicated mice, aged of 10 weeks, were stained with a human-TDP-43 (green) and a ChAT (red) antibodies (*N* = 3). Representative images are shown in **(B)**, and the quantified results are presented in **(C)**. At least 40 motor neurons were scored in each group. The yellow arrow indicates TDP-43 located in the nucleus, and the white arrow indicates TDP-43 mislocalization. Scale bar: 10 μm. **(D)** Relative grip strength was examined after treatment with the indicated drug(s) in A315T TDP-43 mice at the age of 10 weeks. ^*^p < 0.05, significantly different between the indicated groups. n.s., not significant.

Because that D_2_R and A_2A_R were colocalized in motor neurons of the spinal cord (Figure 1B), we next evaluated whether D_2_R also negatively regulated the function of A_2A_R *in vivo*. We treated a TDP-43 (A315T) Tg mice with T1-11 (0.25 mg/ ml in drinking water) in the absence or presence of a D_2_R agonist (quinpirole, 6 mg/kg) from the age of 7 weeks for 3 weeks. It is important to note that the PKA activity of the spinal cord of A315T TDP-43 mice was lower than that of their littermate controls (Figure 4A). Consistent with the *in vitro* studies, chronic treatment with T1-11 elevated the PKA activity in the spinal cord, which was reduced by stimulation of D_2_R using quinpirole, in both A315T TDP-43 mice and their littermate controls (Figure 4A).

We next evaluated the distribution of the transgene (human A315T TDP-43) in the spinal cord of A315T TDP-43 mice using immunofluorescence staining. Localization of motor neurons was marked by expression of ChAT (Figure 4B). No human TDP-43 (hTDP-43) signal was detected in motor neurons of littermate controls (NTG, Figure 4B). Analyses of total TDP-43 (including both endogenous mouse TDP-43 and exogenous hTDP-43) using an anti-TDP-43 antibody that recognized both human and mouse TDP-43 revealed that total TDP-43 was found only in the nuclei of NTG motor neurons (Figure S4A). Conversely, mislocalization of total TDP-43 was observed in motor neurons of A315T TDP-43 mice (Figure S4A), suggesting that the mislocalized TDP-43 was human A315T encoded by the transgene. Analysis of the distribution of human TDP-43 identified by an anti-human TDP-43 antibody demonstrated mislocalization of human TDP-43 (Figure 4B). Chronic treatment with T1-11 for 3 weeks prevented mislocalization of human TDP-43 (Figures 4B,C). Nonetheless, this protective effect of T1-11 on the mislocalization of human TDP-43 was eliminated by co-stimulation with quinpirole (an agonist of D_2_ receptor, Figures 4B,C).

A major phenotype of A315T TDP-43 mice was lower grip strength compared to their littermate controls (Figure 4D). Chronic treatment with T1-11 for 3 weeks improved their grip strength (Figure 4D). Consistent with the negative effects of D_2_R on PKA and TDP-43 mislocalization, activation of D_2_R using quinpirole impeded the T1-11- mediated improvement in the grip strength of A315T TDP-43 mice (Figure 4D). No significant effect of T1-11 or quinpirole on the body weight of A315T TDP-43 mice was observed (Figure S4B). Collectively, these results confirmed that activation of D_2_R interfered with the function of A_2A_R in motor neurons.

## Discussion

Earlier studies have demonstrated that D_2_R and A_2A_R form complexes and reciprocally regulate each other in medium spiny neurons of the striatum (Ferre et al., 1994). Although D_2_R and A_2A_R have been reported to exist in the spinal cord, the potential interaction between these two receptors in motor neurons has remained largely uncharacterized. In the present study, we presented compelling evidence that demonstrated that D_2_R is co-localized and functionally interacts with A_2A_R in mouse and human motor neurons (Figure 1). Previous studies have demonstrated that complex formation between D_2_R and A_2A_R may modulate the binding affinities and G protein-dependent signaling of these two receptors (Ciruela et al., 2004; Fernández-Dueñas et al., 2012; Ferré et al., 2016). We showed in the present study that activation of D_2_R negatively regulates the cAMP/PKA signaling pathway evoked by A_2A_R both *in vitro* (Figure 2) and *in vivo* (Figure 4A, Figure S5) without significantly affecting the binding affinity of T1-11 toward A_2A_R (Table 2). Most importantly, co-stimulation with D_2_R and A_2A_R eliminated the beneficial effects of A_2A_R elicited by a low affinity A_2A_R agonist (T1-11) on TDP-43 mislocalization both *in vitro* (Figure 3) and *in vivo* (Figures 4B,C). Because D_2_R and A_2A_R are drug targets of several diseases (including motor neurodegenerative diseases and psychiatric disorders), the functional interaction between these two receptors and the potential involvement of a drug-drug interaction requires further attention.

To date, treatments for ALS are limited and ineffective. Ample evidence suggests that elevated oxidative stress greatly contributes to ALS pathogenesis (such as TDP-43 mislocalization; Liu et al., 2015a; Scotter et al., 2015). Indeed, the mode of action of Edaravone (a drug that was recently approved by FDA for treating ALS patients) is inhibition of oxidative stress (Lapchak, 2010; Mullard, 2017). This is of great interest because elevated oxidative stress activates AMPK in motor neurons and causes TDP-43 mislocalization, an early event of ALS pathogenesis (Choi et al., 2001; Zmijewski et al., 2010). Abnormal activation of AMPK was observed in motor neurons of the human ALS spinal cord (Liu et al., 2015a), TDP-43 transgenic mice that harbor wild-type human TDP-43 (Liu et al., 2015a) as well as A315T TDP-43 mutants (Coughlan et al., 2016), and SOD1^G93A^ mice (Lim et al., 2012; Perera et al., 2014; Zhao et al., 2015). Deletion of AMPK α2 in the G85R-mSOD1 or M337V-TDP-43 *Caenorhabditis elegans* ALS models also significantly improved their locomotor activity (Lim et al., 2012). Suppression of oxidative stress, which activates AMPK, or activation of the cAMP/PKA pathway, which suppresses AMPK, are known to prevent TDP-43 mislocalization in motor neurons upon encountering stress (Lapchak, 2010; Mullard, 2017). These findings support a detrimental role of ROS/AMPK in ALS pathogenesis.

A_2A_R is a Gαs-coupled receptor that enhances the cellular cAMP content upon stimulation (Chen et al., 2014). Studies from several laboratories suggest that A_2A_R is an important drug target for ALS. We demonstrated that chronic treatment with two A_2A_R agonists (JMF1907 and T1-11) improved motor impairments in mouse models of TDP-43 proteinopathy (Liu et al., 2015a) and Figure 4C of the present study. Consistently, earlier reports suggest that stimulation of A_2A_R using a selective agonist (CGS21680) protects primary motor neurons in a PKA-dependent pathway (Komaki et al., 2012) and delays disease onset in a SOD1 mutant mouse model (Yanpallewar et al., 2012). Conversely, administration of caffeine (a non-selective antagonist of adenosine receptors, including A_2A_R) greatly shortens the life span and disease onset of SOD1 mutant mice (Potenza et al., 2013). Activation of another Gαs-coupled receptor (EP2), which produces cAMP upon stimulation, also protects motor neurons from chronic glutamate toxicity in organotypic spinal cord cultures (Bilak et al., 2004). These studies suggest that enhanced PKA activity in motor neurons is beneficial. One possible function of PKA in combating ALS in motor neurons is to suppress abnormal activation of AMPK, as reported earlier (Liu et al., 2015a). In line with the hypothesis, stimulation of A_2A_R reduced abnormal activation AMPK through a PKA-dependent pathway (Ju et al., 2011; Liu et al., 2015a). We performed experiments to confirm that activation of A_2A_R by T1-11 also suppressed oxidative stress-activated AMPK in NSC34 cells (Figure S3). The effects of T1-11 were mediated by the A_2A_R-PKA pathway because two A_2A_R-selective antagonists [SCH58261, SCH; 8-(3-chlorostyryl) caffeine, CSC] and a PKA inhibitor (H89) all prevented the effect of T1-11 on AMPK phosphorylation at Thr^172^ (Figure S3A). Consistent with the importance of D_2_R in regulating the cAMP/PKA pathway, co-stimulation of D_2_R with two D_2_R agonists (quinpirole or quinelorane) hampered the T1-11-mediated protective effects not only on TDP-43 mislocalization (Figures 3C,D) but also on H_2_O_2_-evoked AMPK activation (Figure S3B). A selective antagonist of D_2_R (L741,626, L74; Bowery et al., 1996) eliminated the effects of quinpirole and quinelorane on AMPK activation (Figure S3B) and TDP-43 mislocalization (Figures 3C,D). Collectively, AMPK may function downstream of PKA to converge the signal from D_2_R and A_2A_R on TDP-43 mislocalization.

Although quinpirole has a higher affinity for D_2_R, quinpirole also activates D_3_R and, therefore, is considered to be a D_2_/D_3_ agonist (Seeman and Van Tol, 1994). In mice, quinpirole at high dosages (5 mg/kg or higher) has been used to investigate the role of D_2_R under pathophysiological conditions (e.g., suppression of neuroinflammation, inhibition of angiogenesis, induction of neurogenesis, and social-emotional reactivity) (Gendreau et al., 1998; Basu et al., 2001; de Haas et al., 2012; Choi et al., 2014; Zhang et al., 2015). With the dosage of quinpirole (6 mg/kg) employed in the present study, we cannot exclude the involvement of D_3_R. Earlier studies have shown that D_3_R also exists in the spinal cord, but its level is much lower than that of D_2_R (Zhu et al., 2007). Moreover, treatment with a selective D_2_R antagonist (L741,626; 10 mg/kg; Bowery et al., 1996) reversed the suppressing effect of quinpirole on the T1-11-mediated normalization of PKA activity in the spinal cord (Figure S5). Collectively, these results suggest that D_2_R is likely the major receptor that negatively regulates the protective effect of A_2A_R. Another interesting aspect is that, similar to D_2_R, D_3_R is also coupled to the Giα protein and inhibits the adenylyl cyclase/cAMP/PKA pathway upon activation (Beaulieu and Gainetdinov, 2011). An earlier study reported that A_2A_R and D_3_R also interact and form a heterodimer (Torvinen et al., 2005). If D_3_R was activated by quinpirole, that interaction may have led to the same suppressing effect on the action of T1-11 as its interaction with D_2_R.

Functional interactions between D_2_R and A_2A_R in the striatum have been extensively investigated in the past few decades (Ongini and Fredholm, 1996; Svenningsson et al., 2000). These D_2_R/A_2A_R complexes not only exist *in vivo* but can also be regulated by pathophysiological activities and stresses (e.g., levodopa-induced dyskinesia, cocaine self-administration, and habit formation; He et al., 2016; Borroto-Escuela et al., 2017; Zhou et al., 2017), as well as appear to modulate the pharmacological characters and signaling pathways of both D_2_R and A_2A_R (Fernández-Dueñas et al., 2012, 2013). In the present study, our findings demonstrate that D_2_R and A_2A_R are co-localized in mouse and human motor neurons and functionally interact at the cAMP/PKA level (Figures 1–3; Table 1). Using a TDP-43 proteinopathy animal model (A315T TDP-43 Tg mouse), we further demonstrated that D_2_R may play a critical role in the protective effects of A_2A_R in ALS (Figure 4). This is important because A_2A_R and/or the cAMP/PKA pathway are potential drug targets for ALS, while agonists and partial agonists of D_2_R have been used in clinics to treat psychiatric disorders (e.g., schizophrenia, gambling disorder, depression; Mété et al., 2016; Earley et al., 2017; Hsu et al., 2017) and psychosis-associated diseases (e.g., Alzheimer's disease, Reeves et al., 2017). As depression and anxiety are commonly observed in ALS patients (Stephens et al., 2016), the potential interaction between A_2A_R agonists and D_2_R agonists in the treatment of ALS patients requires caution and proper judgment. Whether physical interactions between D_2_R and A_2A_R are required for the cross-talk between these two receptors in the motor neurons of spinal cords is of great interest and warrants further investigation.

## Author contributions

C-YL performed the experiments and wrote the manuscript; Y-JL and H-CK provided human motor neurons and analyzed the data; H-LL performed the PKA assays; H-MC conducted the animal studies; Y-PL and YC analyzed the data and wrote the manuscript.

### Conflict of interest statement

YC holds patents in adenosine compounds for the treatment of neurodegenerative diseases. The other authors declare that the research was conducted in the absence of any commercial or financial relationships that could be construed as a potential conflict of interest.
