# Supplementary material for: The D2 Dopamine Receptor Interferes With the Protective Effect of the A2A Adenosine Receptor on TDP-43 Mislocalization in Experimental Models of Motor Neuron Degeneration
Source: Front Neurosci. 2018 Mar 20;12:187. doi: 10.3389/fnins.2018.00187 (PMC5869924; doi:10.3389/fnins.2018.00187)
Supplement: Supplementary file 1 [file Presentation1.pdf]

Supplementary Material

Full title: **The D2 dopamine receptor interferes with the protective effect of the A_{2A} adenosine receptor on TDP-43 mislocalization in experimental models of motor neuron degeneration**

Authors: Chia-You Lai[1, 2], Yu-Ju Liu[2], Hsing-Lin Lai[2], Hui-Mei Chen[2], Hung-Chi Kuo[3], Yu-Ping Liao[2] and Yijuang Chern[1, 2]

Affiliations: [1] Institute of Life Sciences, National Defense Medical Center, Taipei, Taiwan; [2] Institute of Biomedical Sciences, Academia Sinica, Taipei, Taiwan; [3] Institute of Cellular and Organismic Biology, Academia Sinica

Corresponding author:
Yijuang Chern, Institute of Biomedical Sciences, Academia Sinica, Taipei 115, Taiwan
Tel: 886-2-26523913
Fax: 886-2-27829143
E-mail: bmychern@ibms.sinica.edu.tw

Supplementary Material

Receptor binding assay

NSC34 cells were sonicated for 30 seconds on ice with 1 mL of homogenization buffer (25 mM Tris, pH 8.0, 1 mM EGTA, 1 mM $MgCl_2$, 100 μM phenylmethylsulfonyl fluoride, 10 nM okadaic acid, 40 μM leupeptin, EDTA-free Protease Inhibitor and phosStop) and centrifuged at 1000 x g for 10 min at 4 °C. Supernatants were transferred to centrifuge tubes (rotor TLA100.3; BECKMAN) and centrifuged at 50,000 x g for 1 h at 4 °C. Pellets were harvested and suspended in 0.2 mL of lysis buffer (20 mM HEPES, pH 8.0, 0.2 mM $MgCl_2$, 0.2 mM EGTA, 0.1 mM leupeptin, 40 μM phenylmethylsulfonyl fluoride, 30 nM okadaic acid, 0.2 mM sodium orthovanadate, EDTA-free Protease Inhibitor and phosStop). The protein concentrations were determined using the Bradford protein assay (Bio-RAD). Samples were stored at -80 °C. For the binding assays, membranes were incubated with the indicated concentrations of T1-11 in the absence or presence of quinpirole (a D_2R agonist; 1 μM), together with 50 nM of ^{3}H-CGS21680 in a volume of 250 μl of incubation buffer (25 mM HEPES, 140 mM NaCl, 5.4 mM KCl, 1 mM EDTA and 0.3 % BSA, pH 7.4) for 90 min at 25 °C. The inhibition constant (K_i) values were calculated as previously described (Cheng and Prusoff 1973). Excess free ligands were removed using a vacuum filtration manifold with glass microfiber filters GF/C (MERCK Millipore, USA). After extensive washes, the radioactivity of ^{3}H-CGS21680 remaining on the filters was measured by a β counter (BECKMAN, USA).

Animals and drug(s) administration

Wildtype mice were fed with T1-11 (0.25 mg/ml) (Huang, Lin et al. 2011) or vehicle (1% DMSO) in their drinking water. Quinpirole (6 mg/kg; (de Haas, Seddik et al. 2012)) or saline was given to animals by a daily intraperitoneal injection. To assess the involvement of D_2R, a potent D_2-selective antagonist (L741,626, L74, 10 mg/kg, intraperitoneal injection, Tocris; (Bowery, Rothwell et al. 1994)) or vehicle (10% DMSO plus 10% kolliphorEL (Sigma) in saline) was given 10 min before the daily injection of quinpirole.

Supplementary Figure Legends

Fig S1.

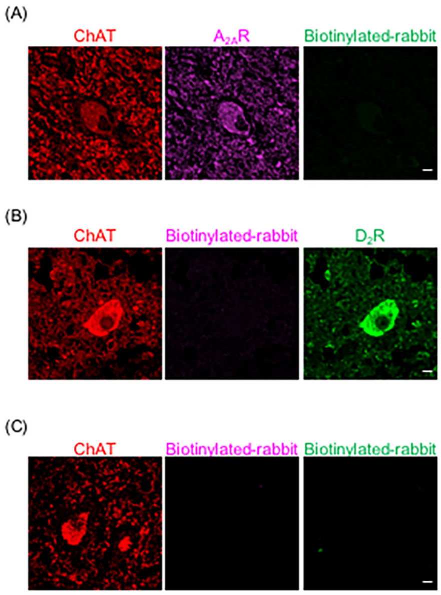

Figure S1. Negative controls of TSA immunostaining with $A_{2A}R$ or D_2R in motor neurons of human spinal cord sections. (A) Human spinal cord sections were stained with an anti-$A_{2A}R$ (purple, A) alone or an anti-D_2R antibody alone (green, B) or no antibodies (C), and a motor neuron marker (ChAT, red), followed by a TSA-amplified immunofluorescence method. Scale bars: 10 μm.

Fig S2.

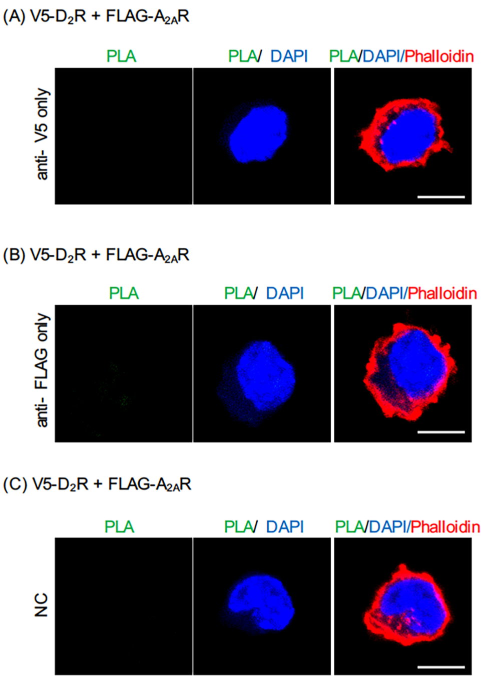

Figure S2. Negative controls of the PLA assay. NSC34 cells were transfected with V5-D_2R and FLAG-$A_{2A}R$ for 48 h. Cells were immunostained with only a V5 antibody (A) or a FLAG antibody (B) or no antibodies (C). NC: negative control. Microfilaments were observed using rhodamine-phalloidin (red). Scale bars: 10 μm.

Fig S3.

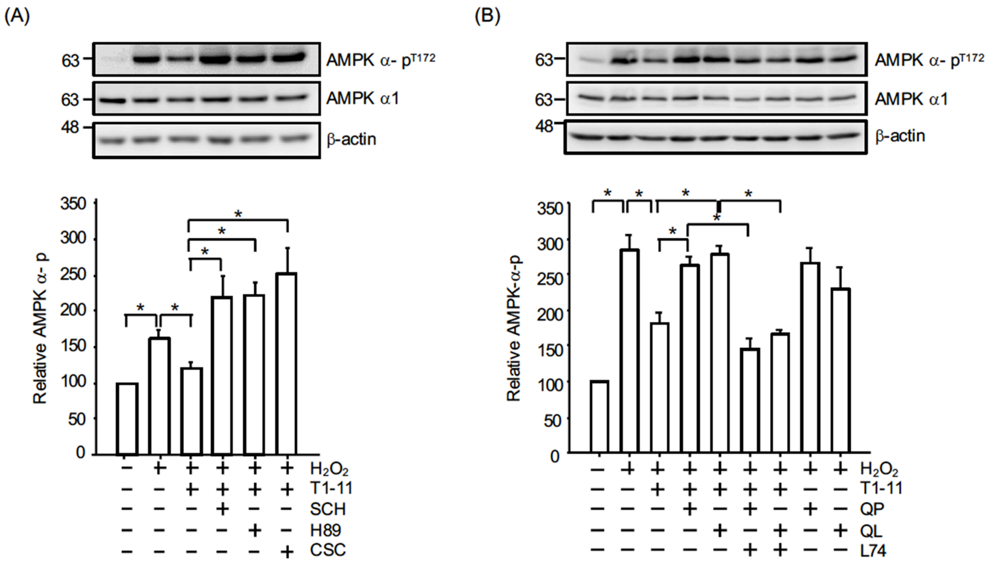

Figure S3. Activation of D_2R suppressed the $A_{2A}R$-mediated inhibition of AMPK activity. (A) NSC34 cells were treated with the indicated drug(s) [H_2O_2, 500 μM; T1-11, 30 μM; SCH58261 (SCH; an $A_{2A}R$ antagonist, 10 μM); H89 (a PKA inhibitor, 5 μM); CSC (an $A_{2A}R$ antagonist, 10 μM)]. (B) NSC34 cells were treated with the indicated drug(s) [H_2O_2, 500 μM; T1-11, 30 μM; QP (1 μM); quinelorane (QL; a D_2R agonist; 1 μM) or L741,626 (L74; a selective D_2R antagonist, 10 μM)] for 4 h, and analyzed for AMPK activation by Western blot analyses. * p <0.05, statistically different between the indicated groups. Data are presented as the mean $\pm$ SEM of three individual experiments.

Fig S4.

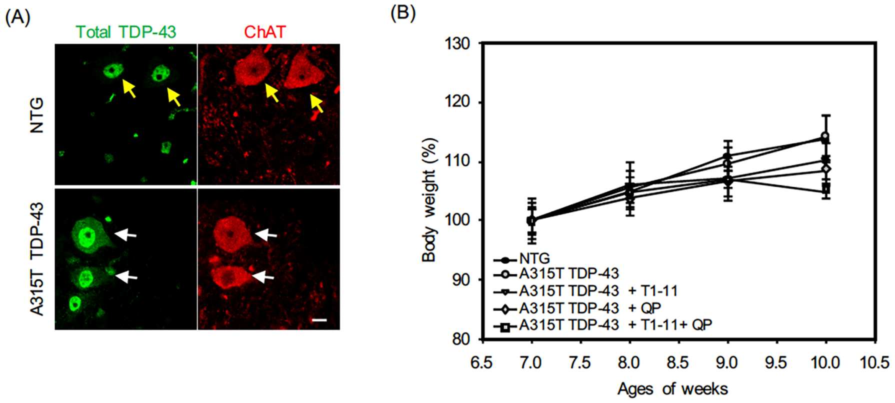

Figure S4. TDP-43 mislocalization and body weight of A315T TDP-43 mice. NTG and A315T TDP-43 were treated with T1-11 (0.25 mg/ml) or vehicle (1 % DMSO) in the drinking water and co-treated with QP (6 mg/kg) or saline by intraperitoneal injection from the age of 7 weeks (N= 5-7). (A) Spinal cord sections of NTG and A315T TDP-43 mice were stained with a mouse/human TDP-43 antibody (green) and a motor neuron marker (ChAT, red) at the age of 10 weeks old. The yellow arrow indicates TDP-43 located in the nucleus. The white arrow marks TDP-43 mislocalization. Scale bars: 10 μm. (B) Body weight of NTG and A315T TDP-43 were measured weekly. Data are presented as the mean $\pm$ SEM.

Fig S5.

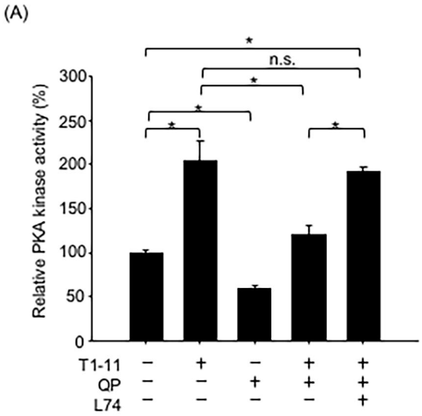

Figure S5. Activation of D_2R inhibited the $A_{2A}R$-mediated PKA activity *in vivo*. (A) Wildtype mice were treated with T1-11 (0.25 mg/ml) or vehicle (1% DMSO) in the drinking water and co-treated with QP (6 mg/kg) or saline by intraperitoneal injection from the age of 7 weeks. To assess the involvement of D_2R, a potent D_2-selective antagonist L741,626 (L74, 10 mg/kg, intraperitoneal injection) or vehicle was given 10 min before the daily injection of QP as indicated. Spinal cords from the indicated mice were harvested to examine PKA activity at the age of 9 weeks (N = 4). * p <0.05, statistically different between the indicated groups. n.s., not significant.

Reference

Cheng, Y. and W. H. Prusoff (1973). "Relationship between the inhibition constant (K1) and the concentration of inhibitor which causes 50 per cent inhibition (I50) of an enzymatic reaction." Biochem Pharmacol 22(23): 3099-3108.
